# Pulmonary Calcifying Fibrous Tumor in a Pediatric Patient: A Case Report

**DOI:** 10.7759/cureus.62053

**Published:** 2024-06-10

**Authors:** Andrés Felipe Herrera Ortiz, Valeria Del Castillo, José N Duarte, María J Gutiérrez, Valeria Noguera, Daniel A Martínez de los Ríos, Sandra P Maldonado Acevedo, Jhon L Torres, Bibiana Pinzón, Angela Moreno, Alejandro J Quiroz Alfaro

**Affiliations:** 1 Department of Radiology, Fundación Santa Fe de Bogotá, Bogotá D.C., COL; 2 Department of Radiology, Universidad El Bosque, Bogotá D.C., COL; 3 Department of Medicine and Health Sciences, Universidad del Rosario, Bogotá D.C., COL; 4 Department of Medicine and Health Sciences, Universidad El Bosque, Bogotá D.C., COL; 5 Department of Internal Medicine, North Mississippi Medical Center, Tupelo, USA

**Keywords:** unusual diagnosis, high-resolution computed tomography, lung hamartoma, pulmonary sequestration, psammomatous calcification, pediatrics, magnetic resonance imaging, inflammatory myofibroblastic tumor, lung neoplasm, calcifying fibrous tumor

## Abstract

A calcifying fibrous tumor (CFT), also known as calcifying fibrous pseudotumor, is an uncommon non-cancerous neoplasm usually located in the gastrointestinal tract. Its location in the lung is extremely rare, and only a few case reports have been published. This case report describes our diagnostic approach in a 9-year-old male patient with an incidental pulmonary mass. The mass was initially misdiagnosed, requiring multiple imaging tests and interventions to obtain the definitive diagnosis of pulmonary CFT. This paper aims to contribute to the limited information available on pulmonary CFT by presenting detailed findings from computed tomography and magnetic resonance imaging.

## Introduction

A calcifying fibrous tumor (CFT) is a rare benign lesion composed of hyalinized collagen with psammomatous calcification, typically showing a pattern of lymphocytic inflammation [1]. This type of lesion typically occurs within soft tissues, with only a few reported cases in children; as a result, its incidence in the pediatric population is currently unknown [1]. Additionally, CFT affecting the lungs has been rarely reported [1]. The cause of CFT is not clear, but it is believed that these lesions may result from a benign inflammatory stimulus [1]. Some authors support that CFTs are the end stage of myofibroblastic inflammatory tumors [2]. There is also limited information on pulmonary CFTs and their findings on diagnostic cross-sectional imaging. 

Here, we present our diagnostic approach in a nine-year-old male patient with an incidental pulmonary mass. The mass was initially misdiagnosed as pulmonary sequestration, giant hamartoma, and fungal infection, requiring multiple imaging tests and interventions before reaching the definitive diagnosis of pulmonary CFT. We also include the detailed findings from the contrast-enhanced high-resolution chest computed tomography (CT) and magnetic resonance imaging (MRI) performed as part of the workup.

## Case presentation

**Figure 1:**
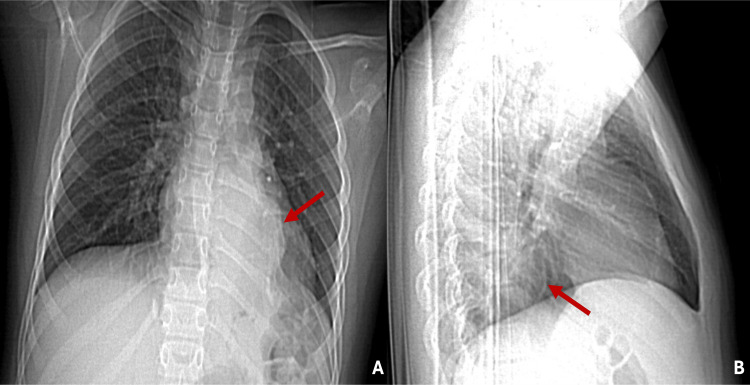
Chest X-ray in posteroanterior and lateral projections showing a retrocardiac intrapulmonary mass in the left lower lung (red arrows in A and B).

A nine-year-old male patient was rushed to the hospital after he fell from his height while playing soccer and presented generalized pain in the ribcage. His vital signs on admission were unremarkable; his physical examination revealed mild pain upon palpation of multiple ribs on the left lateral side of his thorax. The rest of the physical examination was unremarkable, including lung auscultation and neurological examination. Paracetamol was administered for the mild ribcage pain. A chest X-ray was ordered to rule out rib injuries, revealing an incidental pulmonary retrocardiac mass in the left lower lobe, which was initially misdiagnosed as pulmonary sequestration (Figure 1).

**Figure 2:**
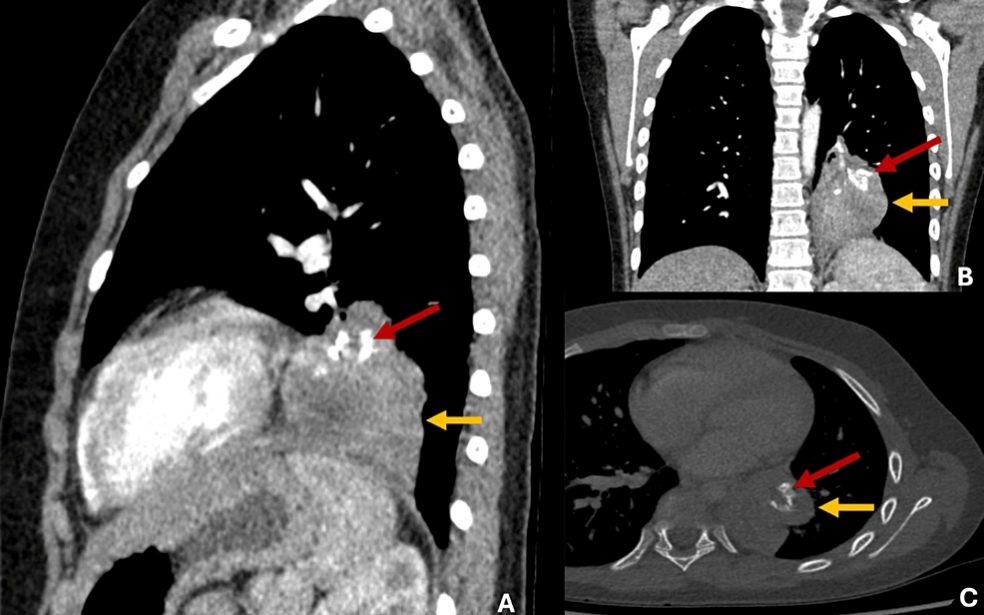
(A-C) Contrast-enhanced high-resolution chest CT reveals an intrapulmonary mass in the left inferior lobe measuring 45 mm x 51 mm. The mass exhibits irregular edges with soft tissue density (yellow arrows) and central calcifications (red arrows). Additionally, there was no evidence of feeders from the thoracic aorta.

To better characterize the lesion, a contrast-enhanced high-resolution chest CT was performed, revealing an intrapulmonary mass with soft tissue density and central calcifications with no arterial supply from the thoracic aorta, ruling out the diagnosis of pulmonary sequestration (Figure 2).

**Figure 3:**
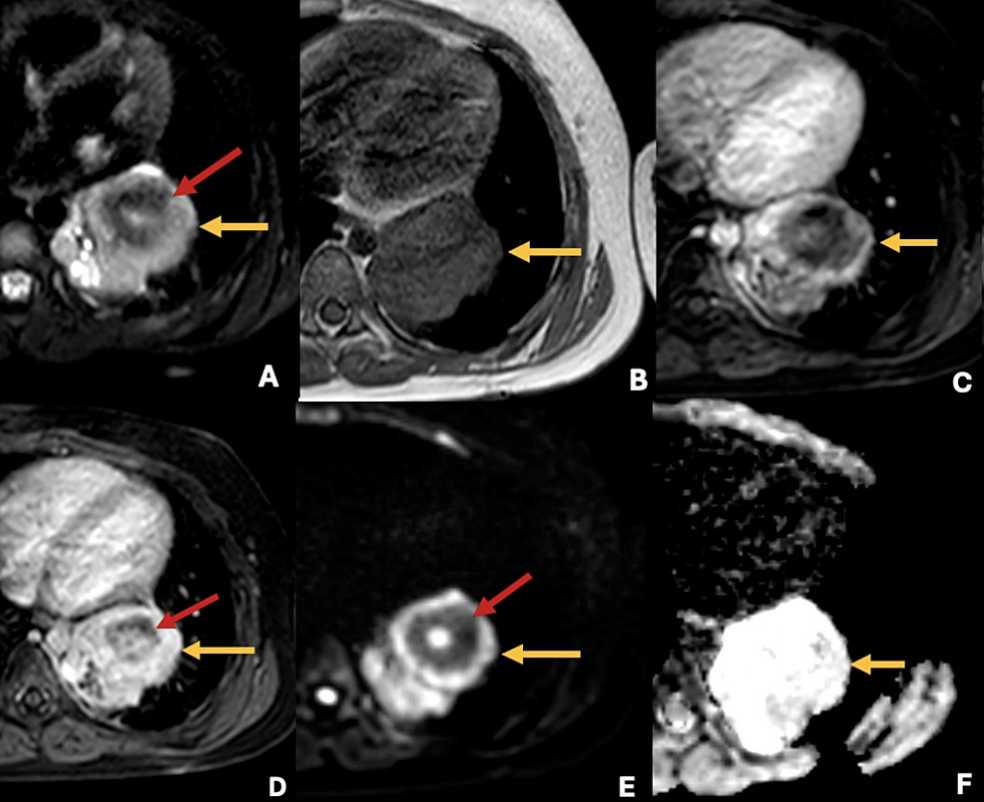
Thoracic MRI shows an intrapulmonary mass with high signal intensity in T2 sequences (yellow arrow in A), low signal intensity in T1 sequences (yellow arrow in B), with late contrast enhancement (yellow arrows in C and D), and no diffuse restriction (yellow arrows in E and F); central calcifications are shown (red arrows).

Based on the CT findings, the differential diagnoses of the lesion were giant hamartoma or fungal infection; therefore, a thoracoscopic biopsy was carried out, reporting inconclusive histopathological findings from an insufficient sample. Due to the inconclusive findings and to further characterize the lung lesion, a thoracic MRI was conducted. The MRI showed a T2 hyperintense and T1 hypointense lesion with central calcifications, no diffuse restriction, and late contrast enhancement, suggesting differential diagnoses of CFT and less likely fungal infections or hamartoma (Figure 3).

To obtain a definitive diagnosis, the patient underwent a left lower lobectomy. Multiple tissue samples from the lung mass were stained with hematoxylin and eosin, showing alveolar tissue consistent with pulmonary parenchyma collapsed by substantial collagen proliferation. Immunohistochemistry was negative for beta-catenin, cluster of differentiation 34 (CD34), acute myeloid leukemia (AML), signal transducer and activator of transcription 6 (STAT6), S100, SOX 10, CKAE1AE3, special at-rich sequence-binding protein 2 (SATB2), B-cell lymphoma 2 (BCL2), immunoglobulin G4 (IgG4), and cyclin-dependent kinase 4 (CDK4). The Ki67 index was less than 1% in the collagen tissue. The histopathology findings were consistent with a CFT. The patient had a smooth postoperative recovery and is currently asymptomatic without evidence of relapse on chest X-rays after a year and a half.

## Discussion

CFT is a rare benign neoplasm found in various anatomical locations. Its pulmonary localization is particularly unusual, with only five cases previously reported in the literature [3,4].

Although its etiology remains unknown, it has been associated with chronic inflammatory processes or trauma [5]. Some authors support that CFTs are the end stage of myofibroblastic inflammatory tumors [2]. Macroscopically, CFTs present as a soft and elastic lesion of grayish color with psammomatous calcifications. Microscopically, the presence of nests of fibrous tissue and hyalinized collagen with low cellularity, along with Psammoma bodies inside, is noteworthy [5,6]. Immunohistochemistry shows focal positivity for the CD34 marker, being negative for anaplastic lymphoma kinase (ALK) [6,7]. A summary of pulmonary CFT cases is shown in Table 1.

**Table 1:** Summary of pulmonary CFT cases. CFT, calcifying fibrous tumor

Case	Age	Gender	Location	Size	Chest CT findings
Peachell et al. (2003) [1]	31	Male	Middle lobe	2.7 cm in diameter	Sharply circumscribed mass with soft tissue density, non-enhancing, with no fat or calcification
Soyer et al. (2004) [8]	7	Male	Right lower lobe	3.1 cm x 3.8 cm x 3.5 cm	Pulmonary calcified mass adherent to inferior vena cava and esophagus
Özkan et al. (2014) [9]	64	Male	Left lower lobe	2.9 cm in diameter	Non-enhancing hypodense mass without calcifications
Zhou et al. (2019) [4]	46	Male	Left lower Lobe	3.5 cm × 2.7 cm × 2.2 cm	Not available
Zhou et al. (2019) [4]	65	Female	Left upper and lower lobes	2 cm × 1 cm × 1 cm, 1 cm × 0.8 cm × 0.5 cm, 0.6 cm × 0.5 cm × 0.5 cm	Not available
Current case	9	Male	Left lower lobe	5.1 cm in diameter	Intrapulmonary mass with soft tissue density, irregular edges, and central calcifications

The age of presentation of pulmonary CFT is variable, with only two cases reported in children. Surprisingly, the data reveal that five out of six pulmonary CFT cases were observed in males; this contradicts the information documented in the literature, which indicates a preference for females in the overall incidence of CFT throughout the body [3]. Almost all cases were reported as solitary lesions, with a mean diameter ranging between 2.5 and 5.1 cm. The case reported by Zhou et al. is the only one with multiple lesions [4]. Pulmonary CFT revealed a predilection for the lower lobes, with one case in the right lower lobe and four in the left lower lobe [4,8,9]. The cases reported by Peachell et al. and Zhou et al. are the only ones in the middle lobe and the upper lobe, respectively [1,4].

Imaging features of CFT usually present as a well-demarcated calcifying mass [10-16]. Calcifications are a distinctive feature [17,18]; however, they can exhibit variable calcification patterns and may even be absent, as in the cases of pulmonary CFT reported by Peachell et al. and Özkan et al. [1,3,9,19]. The enhancement pattern is variable and dependent upon the tumor composition and vascularity [18]. MRI is the preferred modality for characterizing CFT; histological components determine the signal behavior [20,21]. Given their hyalinized collagen tissue, CFTs are characterized by a low signal intensity in T1 and T2 [18]. However, if myxoid stroma predominates, the tumor will feature a high T2 and low T1 signal intensity [22].

The differential diagnoses for a calcified intrapulmonary mass in a pediatric patient encompass a variety of conditions including CFT. Pulmonary sequestration can also present with calcifications; therefore, it is essential to evaluate the blood supply [23]. Hamartoma stands as the second most common benign tumor in children, typically presenting as a well-defined round or lobulated structure with popcorn-like calcifications [24]. Carcinoid tumors are the most prevalent primary malignant lung tumors in older children, typically present as well-defined masses, with calcifications detected in approximately 30% of cases [22,24]. Primary pulmonary tuberculosis can show calcified intrapulmonary lesions and lymph nodes in up to 30% of cases [24,25]. Imaging features of histoplasmosis can manifest as a pulmonary nodule with central calcifications associated with reticulonodular infiltrates and lymphadenopathy [26,27].

Currently, no medical therapy is available for CFT. Surgical resection of the lesion is the definitive treatment [5]. The prognosis for CFT is excellent, with a long-term survival rate of 100% and a recurrence rate of 9%-10% [5,7]. Given the uncommonness of this tumor, case reports are the primary source of evidence. Therefore, we aim to raise awareness about this disease, emphasizing its crucial role as a differential diagnosis when evaluating pulmonary masses in the pediatric population [28].

## Conclusions

This case report deepens into the differential diagnoses of calcified pulmonary masses in pediatric patients. Despite an inconclusive biopsy, thoracic MRI provided crucial insights to diagnose CFT. We hope the insights gained from this case hold significance for both the radiological and surgical communities, offering valuable perspectives for future research and clinical management of similar cases.
